# Mitophagy alleviates neuronal damage after subarachnoid hemorrhage: Role of autophagy-targeting chimera 4

**DOI:** 10.4103/NRR.NRR-D-25-00705

**Published:** 2025-10-30

**Authors:** Yongzhi Zhang, Jianqiao Li, Qi Sun, Peichun Zhou, Pei Wu, Zhiyong Ji, Yuchen Li, Huaizhang Shi

**Affiliations:** 1 Department of Neurosurgery, The First Affiliated Hospital of Harbin Medical University, Harbin, Heilongjiang Province, China; 2 Harbin Medical University, Harbin, Heilongjiang Province, China

**Keywords:** apoptosis, autophagy, brain injury, mitochondrial membrane potential, mitochondrion, mitophagy, neurons, stroke, subarachnoid hemorrhage, ubiquitin

## Abstract

This study investigated the role of autophagy-targeting chimera 4, a novel activator of autophagy that targets mitochondria, in a subarachnoid hemorrhage model. The data demonstrated that in an *in vitro* mitochondrial damage model, autophagy-targeting chimera 4 reversed carbonyl cyanide 3-chlorophenylhydrazone-induced mitochondrial membrane potential collapse and activated mitophagy. In the *in vitro* subarachnoid hemorrhage model, autophagy-targeting chimera 4 improved neuronal proliferation and migration during the acute phase and reduced neuronal apoptosis after subarachnoid hemorrhage. In the *in vivo* subarachnoid hemorrhage model, autophagy-targeting chimera 4 also decreased neuronal apoptosis during the acute phase, improved neurological function, and ultimately reduced long-term neuronal loss. Additionally, increased ring finger protein 144B expression after subarachnoid hemorrhage was associated with poor prognosis, and autophagy-targeting chimera 4 significantly inhibited ring finger protein 144B expression, thereby activating mitophagy and reducing neuronal apoptosis. The results also showed that the mitophagic marker parkin did not exert protective effects during the acute phase after subarachnoid hemorrhage and might be inhibited by ring finger protein 144B. Moreover, parkin inhibition did not interfere with the mitophagic or apoptotic effects of autophagy-targeting chimera 4. These findings not only confirm that autophagy-targeting chimera 4 exerts neuroprotective effects by targeting mitophagy after subarachnoid hemorrhage, but also demonstrate competitive inhibition between ring finger protein 144B and parkin, leading to poor prognosis in the acute phase after subarachnoid hemorrhage.

## Graphical abstract

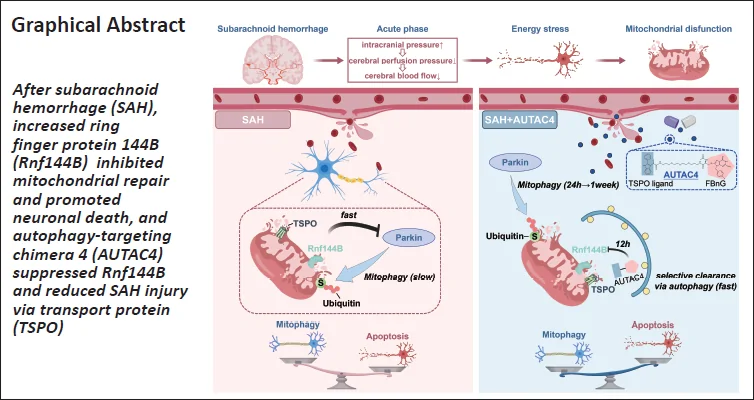

## Introduction

Spontaneous subarachnoid hemorrhage (SAH) is the third most common type of stroke, with aneurysms being responsible for the majority of cases (van Gijn et al., 2007; Claassen and Park, 2022; Kanamaru and Suzuki, 2025; Zhang et al., 2025). The incidence of SAH varies across different populations, with higher rates observed in certain Nordic and Asian countries (van Gijn et al., 2007). There has been a notable decline in the fatality rate of patients who die without reaching a hospital or in the emergency room, which can be attributed to advancements in the early detection and treatment of aneurysms, and improved management strategies (Lawton and Vates, 2017; Claassen and Park, 2022). However, progress in treating complications of SAH, such as early brain injury, elevated intracranial pressure, and delayed cerebral ischemia, has been slow. Currently, nimodipine is the only drug globally recognized to improve prognosis (Allen et al., 1983; Dayyani et al., 2022; Schwarting et al., 2023). Chen et al. (2020) indicated that the meningeal lymphatic system plays a crucial role in clearing erythrocytes from the cerebrospinal fluid after SAH. However, further research is needed to assess the efficacy of targeting this system for therapeutic purposes.

The brain is the most energy-intensive organ, and neurons are heavily dependent on mitochondria for energy to maintain normal function (Dienel, 2019). After SAH, direct stimulation of neurons and strategies to address ischemia, hypoxia, and hemoglobin content can cause indirect damage through the production of degradation products and the subsequent collapse of mitochondrial membrane potential. This collapse leads to the overproduction of reactive oxygen species, the release of apoptotic proteins, and the activation of mitochondria-associated inflammation, all of which further damage neurons and promote the overactivation of microglia (Zhang et al., 2024). Although eukaryotic cells have evolved various mitochondrial quality control mechanisms, including mitophagy, mitochondrial biogenesis and degradation, and mitochondrial fusion and fission (Liu et al., 2024), SAH does not involve a slow dynamic adaptation process as seen with ischemic stroke and tumors (Pickles et al., 2018; Wang et al., 2023). The sudden and severe onset of SAH often leads to the collapse of mitochondrial quality control mechanisms, necessitating external intervention to accelerate recovery (Macdonald, 2014; Robba et al., 2024). Therefore, salvaging neuronal mitochondria has potential to improve the prognosis of patients with SAH (Zhang et al., 2022a).


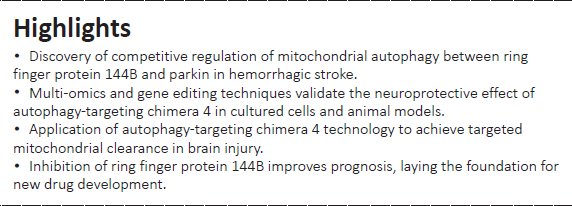


Mitophagy specifically targets mitochondria for elimination and regulates the quality and quantity of these organelles, thereby removing dysfunctional or excessive mitochondria that can produce reactive oxygen species and cause cell death (Guan et al., 2018). However, mitophagy activation is insufficient after SAH, resulting in an excessively fissioned state that leads to neuronal apoptosis and worsens prognosis (Wu et al., 2017; Zhang et al., 2022b). Previous studies of mitophagy have been limited by the lack of any drug capable of specifically triggering this process. For example, the antioxidant properties of mitoquinone activate mitophagy (Zhang et al., 2019), and metformin activates mitophagy by modulating energy metabolism through adenosine 5′-monophosphate-activated protein kinase (Zhang et al., 2022b). Additionally, arachidonyl-2-chloroethylamide indirectly activates mitophagy through targeted activation of cannabinoid receptor 1 (Liu et al., 2022). Moreover, MF094 triggers mitophagy by suppressing ubiquitin-specific protease 30, although its effectiveness is limited by upstream parkin ubiquitination levels and insufficient parkin initiation after SAH (Liu et al., 2023). Therefore, identifying a method or drug to target and activate mitophagy after SAH will help address this current research dilemma.

Takahashi et al. (2019) developed autophagy-targeting chimera (AUTAC) molecules that selectively target specific proteins and dysfunctional mitochondria for autophagy, providing a new direction for drug development. The AUTAC4 molecule consists of three components: (1) a degradation tag of p-fluorobenzylguanine that induces K63-linked polyubiquitination by mimicking the S-guanylation modification, allowing recognition by p62 and interaction with LC3, which ultimately fuses with lysosomes for degradation to trigger autophagy; (2) a polyethylene glycol linker that connects the p-fluorobenzylguanine tag to a mitochondrial ligand at the other end; and (3) a mitochondria-targeting binder (2-phenylindole derivative) that specifically recognizes and binds to the 18-kDa outer mitochondrial membrane transport protein, which has been positively correlated with SAH (Rupprecht et al., 2010; Zhou et al., 2020). Therefore, AUTAC4 can theoretically bind to damaged mitochondria with high transport protein expression after SAH, and thereby eliminate the damaged mitochondria via autophagy. The aims of this study were (1) to determine whether AUTAC4 reduces neuronal damage to improve prognosis after SAH and (2) to elucidate the potential mechanisms underlying the protective effects of AUTAC4 and validate downstream targets.

## Methods

### Animal preparation and spontaneous subarachnoid hemorrhage induction *in vivo*

The study protocol was approved by the Institutional Animal Care and Use Committee of the First Affiliated Hospital of Harbin Medical University, China (approval No. 2024042, approval date: April 26, 2024) and conducted in accordance with the National Institutes of Health Guide for the Care and Use of Laboratory Animals (8^th^ ed., National Research Council, 2011).

Male Sprague–Dawley rats (*n* = 90; body weight: 250–300 g; age: 7–9 weeks; grade: specific pathogen-free) were purchased from Jiangsu Huachuang Sino Biotechnology Co., Ltd. (SCXK (Su) 2020-0009, Jiangsu, China) and were housed in an animal care facility maintained at a constant temperature of 23 ± 2°C, with a 12-hour light/dark cycle and free access to food and water.

The rats underwent endovascular perforation to establish the SAH model (Liu et al., 2022). Briefly, under general anesthesia with pentobarbital sodium, a pointed 4-0 nylon suture was inserted from the stump of the external carotid artery to the internal carotid artery until resistance was felt. The suture was advanced to puncture the artery and then promptly removed. For the rats in the sham group, the same procedures were performed without arterial perforation. At 24 hours or 1 week after SAH, the rats were humanely euthanized with pentobarbital sodium (100 mg/kg) and perfused with cold normal saline before the extraction of the whole brain, which was preserved in liquid nitrogen for further experiments.

### Experimental design

**Figure 1:**
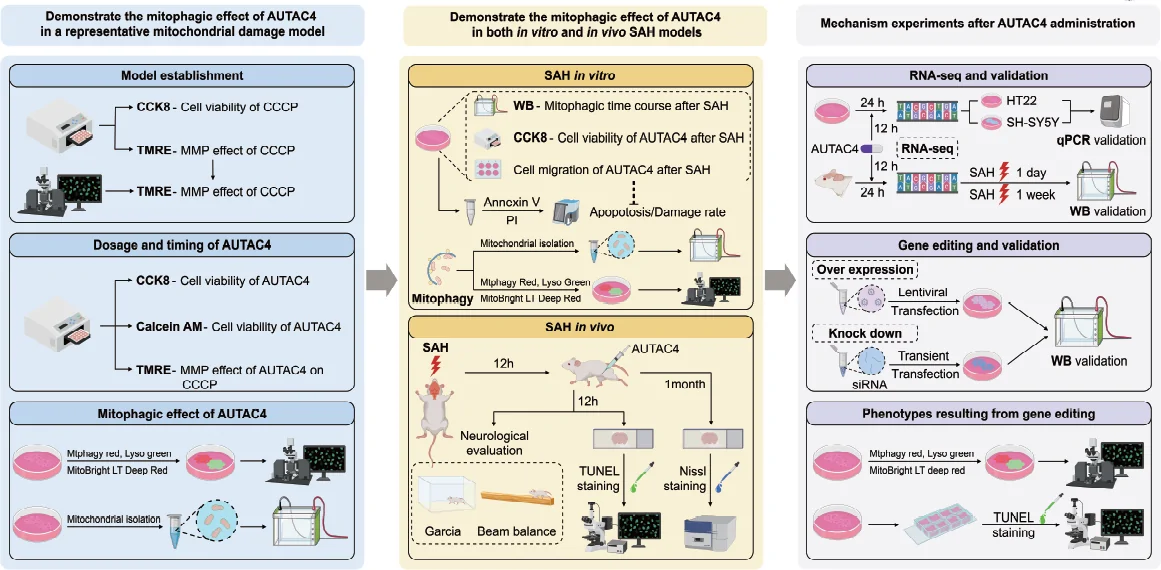
Experimental design. AUTAC4: Autophagy-targeting chimera 4; CCCP: carbonyl cyanide 3-chlorophenylhydrazone; CCK: cell counting kit; FITC: fluorescein isothiocyanate; MMP: mitochondrial membrane potential; PCR: polymerase chain reaction; PI: propidium iodide; RNA-seq: ribonucleic acid sequencing; SAH: subarachnoid hemorrhage; siRNA: small interfering ribonucleic acid; TMRE: tetramethylrhodamine ethyl ester; TUNEL: terminal deoxynucleotidyl transferase dUTP nick end labeling; WB: western blot.

The procedures for all experiments are illustrated in **Figure 1**.

#### Experiment 1

To establish a model of mitochondrial damage, seven groups of HT22 cells (*n* = 6) were treated with various concentrations of carbonyl cyanide 3-chlorophenylhydrazone (CCCP; HY-100941; purity: 98.83%; MedChemExpress, Shanghai, China) at 0 (control), 0.1, 1, 10, 100, 1000, and 10 000 μM, with stimulation durations of 15, 30, 60, and 120 minutes. Cell viability following stimulation with CCCP was assessed using the Cell Counting Kit-8 (CCK8) assay (C6005; NCM Biotech, Suzhou, China). Subsequently, four groups (*n* = 6) were evaluated with CCCP concentrations of 0 (control), 0.1, 1, and 10 μM, and stimulation durations of 15, 30, and 60 minutes. The mitochondrial membrane potential was measured using a tetramethylrhodamine ethyl ester (TMRE) kit (C2001S; Beyotime Biotechnology, Shanghai, China) (Ni et al., 2022). Finally, two groups (*n* = 3) were assessed with CCCP concentrations of 0 (control) and 10 μM, and a stimulation duration of 30 minutes. The mitochondrial membrane potential was then assessed using the TMRE method.

#### Experiment 2

After establishing the mitochondrial damage model, the dosage and timing of AUTAC4 administration were determined. First, six groups (*n* = 6) were established with AUTAC4 concentrations of 0 (control), 1, 10, 25, 50, and 100 μM, and stimulation durations of 3, 6, 12, and 24 hours. The CCK8 assay was performed to evaluate the effects of different concentrations and stimulation times of AUTAC4 on cell viability. Subsequently, five experimental groups (*n* = 6) were established: control, CCCP, and CCCP + AUTAC4 at 1, 10, and 25 μM. AUTAC4 was administered in advance, with stimulation durations of 3, 6, 12, and 24 hours. CCCP (10 µM) was applied for 30 minutes before detection. The Calcein AM Cell Viability Assay Kit (C2013; Beyotime Biotechnology) was used to determine the optimal dose and timing of AUTAC4 administration, and the TMRE method was performed for secondary validation of the AUTAC4 dosage at 12 hours.

#### Experiment 3

After confirming that AUTAC4 alleviates CCCP-induced mitochondrial damage, three groups (*n* = 3) were established: control, CCCP, and CCCP + AUTAC4. AUTAC4 (10 µM) was administered in advance, followed by the addition of 10 μM CCCP 30 minutes later. Live cell mitophagic staining and western blot assays were performed to demonstrate the mitophagic effect of AUTAC4.

#### Experiment 4

To demonstrate the role of AUTAC4 in mitophagy after SAH *in vitro*, a control group was established, along with five groups (*n* = 6) treated with oxyhemoglobin (oxyHb; Sigma-Aldrich, St. Louis, MO, USA) at 3, 6, 12, 24, and 72 hours. Western blot analysis was performed to assess the levels of mitophagic proteins and downstream proteins of AUTAC4 at different time points after SAH. Subsequently, six groups (*n* = 6) were established: control, oxyHb, oxyHb + dimethyl sulfoxide (DMSO; D2650; Sigma-Aldrich), and oxyHb + AUTAC4 at 1, 10, and 25 μM. OxyHb was administered for 24 hours, followed by AUTAC4 stimulation for 12 hours (at 12 hours after oxyHb stimulation). The CCK8 assay was used to assess the effect of AUTAC4 on cell viability after SAH. Subsequently, three groups (*n* = 3) were established: control, oxyHb, and oxyHb + AUTAC4. OxyHb was administered for 24 hours, followed by 10 μM AUTAC4 stimulation for 12 hours (at 12 hours after oxyHb stimulation). HT22 cell migration and apoptosis assays were conducted to validate the effects of AUTAC4 on oxyHb. Finally, two groups (*n* = 3) were established: oxyHb and oxyHb + AUTAC4. Western blot analysis and live cell mitophagic staining were conducted to demonstrate the mitophagic effect of AUTAC4.

#### Experiment 5

To determine the role of AUTAC4 after SAH *in vivo*, five groups (*n* = 6) were established: Sham, SAH + vehicle, SAH + AUTAC4 300 μg/kg, SAH + AUTAC4 1 mg/kg, and SAH + AUTAC4 3 mg/kg. AUTAC4 was administered via intraperitoneal injection 12 hours after SAH, and the optimal dose of AUTAC4 was determined based on neurological function scores at 24 hours post-SAH. Subsequently, three additional groups (*n* = 3) were established: Sham, SAH, and SAH + AUTAC4. Terminal deoxynucleotidyl transferase dUTP nick end labeling (TUNEL) staining was performed 24 hours after SAH to assess neuronal apoptosis, and Nissl staining (C0117; Beyotime Biotechnology) was conducted 30 days after SAH to evaluate neuronal loss.

#### Experiment 6

To identify the downstream targets of AUTAC4 after SAH, three *in vitro* groups (*n* = 3) were established: control, oxyHb, and oxyHb + AUTAC4. Ribonucleic acid sequencing (RNA-seq) was used to screen for differentially expressed genes. Subsequently, the oxyHb and oxyHb + AUTAC4 groups (*n* = 3) were validated using two different cell lines (HT22 and SH-SY5Y) to verify the *in vitro* transcriptomic results. Following this, *in vivo* Sham and SAH groups (*n* = 3) were established, and the results were combined with the *in vitro* transcriptomic analysis. Finally, western blot analysis was performed to verify the selected differentially expressed genes. For *in vitro* validation, six time points (0, 3, 6, 12, 24, and 72 hours; *n* = 6) were examined. *In vivo* validation included three groups (Sham, SAH at 24 hours, and SAH at 1 week; *n* = 3).

#### Experiment 7

To validate the roles of ring finger protein 144B (Rnf144B) and parkin after SAH, five groups (*n* = 6) were established: Hb, Hb + AUTAC4, Hb + AUTAC4 (siParkin), Hb + AUTAC4 (siRnf144B), and Hb + AUTAC4 (Rnf144B-Lentivirus, LV). Western blot analysis was performed to verify the gene editing effects. Subsequently, four groups (*n* = 5) were established: Hb + AUTAC4, Hb + AUTAC4 (siParkin), Hb + AUTAC4 (siRnf144B), and Hb + AUTAC4 (Rnf144B-LV). Intergroup differences were assessed using TUNEL staining and live cell mitophagic staining.

### Cell culture and *in vitro* subarachnoid hemorrhage model

HT22 cells, a mouse hippocampal neuronal cell line (CL-0697; Procell Life Science & Technology Co., Ltd., Wuhan, China), and SH-SY5Y cells, a human neuroblastoma cell line (H8-0101, Oricell Therapeutics Co., Ltd., Shanghai, China), were cultured in Dulbecco’s modified Eagle’s medium (DMEM; Gibco, Thermo Fisher Scientific, Waltham, MA, USA) supplemented with 10% fetal bovine serum (164210; Procell Life Science & Technology Co., Ltd.) and 1% penicillin/streptomycin (C100C5; NCM Biotech, Suzhou, China) at 37°C and in 5% CO_2_/95% air (Shao et al., 2023). As described in a previous study (Wu et al., 2024), 10 μM oxyHb was added to the culture medium for 24 hours to induce SAH *in vitro*.

### Cell viability assay

The viability of HT22 cells was assessed using the CCK8 assay. Preliminary experiments demonstrated that the average optical density of HT22 cells (8 × 10^7^ cells per liter in 100 μL of CCK8 reagent) reached 1.0 after 3 hours of incubation. Therefore, this concentration of cells was added to the wells of 96-well plates. Six replicates of each group were included in the CCK8 assay. Each plate also contained a blank negative control group (complete culture medium without cells) and a positive control group (complete culture medium with cells). After the cells had attached to the plate, the experimental groups were stimulated and then washed once with phosphate-buffered saline (PBS). Subsequently, CCK8 medium (100 μL medium + 10 μL CCK8) was added to each well, and incubation was continued at 37°C for 3 hours. The optical density at 450 nm was measured using a multifunctional microplate reader (Synergy H1; BioTek Instruments, Inc., Winooski, VT, USA).

To detect the effects of different concentrations of AUTAC4 (HY-134640, purity: 98.22%, MedChemExpress, Shanghai, China) on CCCP more sensitively, the Calcein AM Cell Viability Assay Kit was used to assess cell viability (Wang et al., 2024). HT22 cells were added to the wells of 96-well plates with all-black transparent bottoms, containing 100 μL of Calcein AM working solution, and were stimulated in the dark at 37°C for 30 minutes. Finally, the optical density was measured at excitation and emission wavelengths of 490 nm and 520 nm, respectively, using a multifunctional microplate reader (Synergy H1; BioTek Instruments, Inc.).

### Cell migration assay

The scratch assay was performed to assess the migration of HT22 cells. Cells were seeded in the wells of 6-well plates and grown to confluence. A sterile 200-μL pipette tip was used to scratch the cell layer. After washing three times with PBS, cells in the control group were cultured in DMEM, whereas those in the oxyHb and oxyHb + AUTAC4 groups were cultured in DMEM containing 10 μM oxyHb and DMEM containing both 10 μM oxyHb and 10 μM AUTAC4 (12 hours after oxyHb), respectively. At 24 and 48 hours after scratching, the cells were photographed. The area of cell migration was measured using FIJI software (version 2.16.0/1.54p; https://imagej.net/software/fiji/).

### Mitochondrial membrane potential assay

A TMRE Kit was used to detect the mitochondrial membrane potential using a fluorescence microplate reader (Synergy H1) and qualitatively with a laser confocal microscope (LSM980/880; Carl Zeiss AG, Oberkochen, Germany). Six replicates of each group were included for quantitative analysis, along with a blank negative control group (TMRE without cells) and a positive control group (TMRE with cells). HT22 cells (8 × 10^4^/mL in 100 μL of medium) were added to the wells of 96-well plates with all-black transparent bottoms. After attaching to the plate, the cells were stimulated, washed once with PBS, and incubated with TMRE working solution (1 mL TMRE buffer + 1 μL TMRE 1000×) at 37°C for 30 minutes. After washing twice with culture medium, the optical density was measured at excitation and emission wavelengths of 550 nm and 575 nm, respectively, using a multifunctional microplate reader (Synergy H1; BioTek Instruments, Inc.).

For qualitative analysis, HT22 cells were cultured in 35-mm glass-bottom confocal dishes and stimulated after adhesion. Subsequently, the cells were washed once with PBS, and 1 mL of a staining solution containing TMRE and Hoechst 33342 (C1027; Beyotime Biotechnology) was added to each dish. After incubation in the dark at 37°C for 30 minutes, the cells were washed twice with culture medium and assessed using a laser confocal microscope (LSM980/880; Carl Zeiss AG).

### Detection of mitophagy

Mtphagy dye (MD01; Dojindo Laboratories, Kumamoto, Japan) was used to assess mitophagy. When mitochondrial autophagy occurs, damaged mitochondria bind to lysosomes, resulting in an acidic pH that causes the Mtphagy dye to produce strong fluorescence. A working staining solution containing Mtphagy dye with MitoBright LT Deep Red (MT12; Dojindo Laboratories) and LysoPrime Green (L261; Dojindo Laboratories) in DMEM was prepared to identify mitochondria and lysosomes, respectively. After removing the culture medium, HT22 cells were incubated with the staining solution at 37°C for 30 minutes in the dark, followed by two washes with DMEM. After replacing the culture medium, the cells were stimulated for the appropriate duration. Images were obtained using a laser confocal microscope (LSM980/880; Carl Zeiss AG). The laser wavelengths for Mtphagy dye, MitoBright LT Deep Red, and LysoPrime Green were 594 nm, 639 nm, and 488 nm, respectively.

### Mitochondrial isolation

A Cell & Tissue Mitochondria Isolation Kit (C3601/C3606; Beyotime Biotechnology) was used to collect mitochondria for western blot analysis according to the manufacturer’s instructions. In brief, HT22 cells in the CCCP, CCCP + AUTAC4, oxyHb, and oxyHb + AUTAC4 groups were first subjected to modeling and stimulation. The homogenized cells were then centrifuged at 11,000 × *g* for 10 minutes at 4°C. The supernatant contained the cytosolic proteins, and the precipitate contained the mitochondrial fraction. Depending on the number of cells or the weight of brain tissue, a corresponding volume of mitochondrial lysate was added for subsequent analysis. The lysate was then subjected to western blot analysis.

### Flow cytometry

The proportion of apoptotic neuronal cells was detected using an Annexin V-Fluorescein Isothiocyanate (FITC) Apoptosis Detection Kit (C1062; Beyotime Biotechnology) according to the manufacturer’s instructions. In brief, HT22 cells in the control, oxyHb, and oxyHb + AUTAC4 groups were washed once with PBS, digested, and then resuspended in PBS after stimulation. The cell suspension was incubated with Annexin V-FITC binding solution, Annexin V-FITC, and propidium iodide (PI) for 10 minutes in the dark. Half of the cells in the compensation group were heated to 70°C in a metal bath for 5 minutes. Annexin V-FITC binding solution was used for the compensation group, whereas the FITC monochromic staining group was treated with both Annexin V-FITC binding solution and Annexin V-FITC. The PI monochromic staining group received Annexin V-FITC binding solution and PI. FITC and PI were detected at a wavelength of 488 nm using a flow cytometer (Apogee Flow Systems Ltd., Hertfordshire, UK). After detection, FlowJo 10.9 software (BD Biosciences, Franklin Lakes, NJ, USA) was used for statistical analysis and to generate graphs.

### Frozen section preparation

Rat brain tissue was immersed in 4% paraformaldehyde for at least 24 hours. Subsequently, the tissue was soaked in a 30% sucrose solution for at least 48 hours until it sank to the bottom. The dehydrated brain tissue was then placed in a cryosectioning box, and an appropriate amount of liquid nitrogen was poured in to rapidly freeze the tissue for approximately 10 to 20 seconds. The frozen tissue was then transferred to a histology cassette, and pre-cooled optimal cutting temperature compound (C0171A; Beyotime Biotechnology) was added to completely submerge the brain tissue. The entire histology cassette was then placed in a −80°C freezer for 1 hour, after which sectioning was performed on a cryostat at a thickness of approximately 8 μm.

### Terminal deoxynucleotidyl transferase dUTP nick end labeling assay

After completing the modeling and intervention experiments, the proportion of apoptotic neurons was semiquantitatively analyzed using the TUNEL Apoptosis Assay Kit (C1090; Beyotime Biotechnology) both *in vivo* and *in vitro*. Briefly, frozen slices or HT22 cells in the Hb + AUTAC4, Hb + AUTAC4 (siParkin), Hb + AUTAC4 (siRnf144B), and Hb + AUTAC4 (Rnf144B-LV) groups were fixed with 4% paraformaldehyde for 30 minutes and then incubated in 50 μL of TUNEL working solution at 37°C for 1 hour in the dark. Before TUNEL staining, the tissue slices were incubated overnight at 4°C with an anti-NeuN primary antibody, followed by incubation with a fluorescence secondary antibody at 37°C for 1 hour in the dark. The samples were then sealed with mounting medium containing 4′,6-diamidino-2-phenylindole (ab104139; Abcam, Cambridge, UK). Images were captured using a fluorescence microscope (EVOS M5000; Thermo Fisher Scientific).

### Neurological evaluation

Neurological deficits in rats at 24 hours after SAH were evaluated using the Garcia and beam balance scores (Xu et al., 2021; Tian et al., 2022). Briefly, the Garcia score consists of six tests: spontaneous activity, symmetry in movement of the four limbs, forepaw outstretching, climbing, body proprioception, and response to vibrissae touch. The beam balance score was assessed on a scale score from 0 to 4 based on the animals’ ability to walk along a narrow wooden beam for 1 minute, with higher scores indicating better neurological outcomes. Detailed information on the neurological evaluation is provided in **Additional Table 1**.

**Additional Table 1:** Neurological evaluation *Garcia score*

Test	Score			
	0	1	2	3
**Body proprioception**	Not applicable	No response	Weak response	Normal
**Climbing**	Not applicable	Fails to climb	Climbs weakly	Normal climbing
**Forepaw outstretching**	No movement	Slight outreaching	Outreach is limited and less than pre- SAH	Outreach same as pre-SAH
**Response to vibrissae touch**	Not applicable	No response	Weak response	Normal
**Spontaneous Activity (in a cage for 5 min)**	No movement	Barely moves position	Moves but does not approach at least 3 sides of the cage	Moves and approaches at least 3 sides of the cage
**Symmetry in movement of the four limbs**	No movement	Slight movement of limbs	Moves all limbs but slowly	Move all limbs same as pre-SAH
***Beam balance test***				
**Score**	**Beam walking (1 min)**			

**0**	No walking and falls off			
**1**	No walking but remains on beam			
**2**	Walking but falls off			
**3**	Walking ≤ 20 cm			
**4**	Walking > 20 cm			

SAH: Subarachnoid hemorrhage.

### Nissl staining

Hippocampal neuron degeneration at 1 month after SAH was evaluated using Nissl staining. Briefly, frozen slices were fixed with 4% paraformaldehyde for 10 minutes, incubated with Nissl staining solution for 10 minutes, and then dehydrated twice with 95% ethanol and cleared twice with xylene. Images were captured using a slide scanner (VS200; Olympus Corporation, Tokyo, Japan).

### Quantitative polymerase chain reaction and RNA-seq analyses

After completing the modeling and intervention experiments, total RNA from each group was isolated immediately using the NcmSpin Cell/Tissue Total RNA Kit (M5105; NCM Biotech, Suzhou, China) according to the manufacturer’s instructions. RNA was reverse-transcribed into complementary deoxyribonucleic acid (cDNA) using the ReverTra® Ace qPCR RT Kit (FSQ-101; Toyobo Co., Ltd., Osaka, Japan). The SYBR method was used to amplify cDNA with gene-specific primers (Comate Bioscience Co., Ltd., Changchun, China) (see **Additional Table 2**) and detection was performed using a real-time fluorescence quantitative PCR instrument (LightCycler® 480 II; Roche, Basel, Switzerland). Glyceraldehyde-3-phosphate dehydrogenase (GAPDH) was used as an internal reference for the relative quantification of each target gene. The differences in target gene expression between groups are presented as fold changes.

**Additional Table 2:** Primer sequences

Gene	Primer sequence	Product size (bp)
GAPDH-human	5’-GTC TCC TCT GAC TTC AAC AGC G-3’ 5’-ACC ACC CTG TTG CTG TAG CCA A-3’	131
GAPDH-mouse	5’-CAT CAC TGC CAC CCA GAA GAC TG-3’ 5’-ATG CCA GTG AGC TTC CCG TTC AG-3’	153
MAB21L3-human	5’-AGG CAC CTG AAG GAG GAC ATC T-3’ 5’-GCT TTG CTG AAG ACC TGC CAG T-3’	131
Mab21l3-mouse	5’-GCT AAC CAC AGA AAT CAG CCG C-3’ 5’-GCC TTT CAG TGT GAC GGT GAC A-3’	124
ND4L-human	5’-CCC TCG TAG TAA CAG CCA TTC TC-3’ 5’-CGA CTG TGA GTG CGT TCG TAG T-3’	133
Pram1-human	5’-CCG TGG ATA TGC AGA GCT TTC G-3’ 5’-GGG TTC CAC ATC GTC ATA CAG C-3’	155
Pram1-mouse	5’-GAA GAC AGG CTC TTC TAC TGG G-3’ 5’-GGC TGA TAC TAG AGT CAT CTG TG-3’	125
Rnf144B-human	5’-GCA AAC TCT GCC TGT GTG AGC A-3’ 5’-CAT CCT TCT CGG ATT GCC AGC T-3’	118
Rnf144B-mouse	5’-TGG TGC TGT CAA TCC GAG AAG G-3’ 5’-CAA ACT TGA GCC GCT GAT ACA GC-3’	144
SRP54-human	5’-GTC AGT TTA CGT TGC GAG ACA TG-3’ 5’-GCC TTG CCA TTG ACT CCT GTT C-3’	141
Srp54a-mouse	5’-AAA GCG TGG ACA CCG ACT AAG G-3’ 5’-GGT CTT CCA ACC TTT CCT CTG G-3’	123
ZNF17-human	5’-GAC GTT CAG AGA CAC CTG CAC A-3’ 5’-CAC ATT GCT TGG AAG GTG CCT C-3’	112

GAPDH: Glyceraldehyde-3-phosphate dehydrogenase; Mab21l3: mab 21 like 3; ND4L: NADH dehydrogenase subunit 4L; Pram: PML-RARA regulated adaptor molecule; Rnf: ring finger protein; SRP: signal recognition particle; ZNF: zinc finger.

For RNA-seq analysis, total RNA was extracted from HT22 cells or brain tissue using TRIzol reagent (15596026CN, Thermo Fisher Scientific). Subsequently, messenger RNA enrichment and fragmentation, reverse transcription, cDNA repair, amplification, purification, and library construction were performed by Novogene (Beijing, China).

### Transfection and lentivirus-mediated gene overexpression

Transfection of HT22 cells was performed using the LipoRNAi^TM^ Transfection Reagent (C0535; Beyotime Biotechnology) according to the manufacturer’s instructions. Briefly, when the cell density reached approximately 70%, a mixture of DMEM, small interfering RNA (siRNA), and LipoRNAi™ was added to the medium, and incubation was continued for 48 hours. The siRNA sequences are listed in **Additional Table 3**. After verifying the transfection efficiency through western blot analysis, the cells were continuously cultured and subjected to modeling experiments.

**Additional Table 3:** siRNA sequences

siRNA	Sequence	Product size (bp)
Parkin-mouse- 81- siRNA 1	5’-GGA AGU GGU UGC UAA GCG ATT-3 ’ 5’-UCG CUU AGC AAC CAC UUC CTT-3’	21
Parkin-mouse-919-siRNA2	5’-GGA GAA GAG CAG UAC ACU ATT-3 ’ 5’-UAG UGU ACU GCU CUU CUC CTT-3’	21
Parkin-mouse-224-siRNA3	5’-GGA GAA GUC AUG AAA CAA ATT-3 ’ 5’-UUU GUU UAA UGA CUU CUC CTT-3’	21
Rnf144b-mouse-152- siRNA 1	5’-GGC UGU GGA UCU CCU AUC ATT-3’ 5’-UGA UAG GAG AUC CAC AGC CTT-3’	21
Rnf144b-mouse-616-siRNA2	5’-GAA CCU GGA CAA UGA CAU ATT-3’ 5’-UAU GUC AUU GUC CAG GUU CTT-3’	21
Rnf144b-mouse-241- siRNA3	5’-GGA UGA GUU UCA GCU GUA UTT-3’ 5’-AUA CAG CUG AAA CUC AUC CTT-3’	21

Rnf: Ring finger protein; RNA: ribonucleic acid.

Stable HT22 cells overexpressing Rnf144B were constructed via lentiviral transfection. The viral vector was designed and produced by Cyagen Biosciences Inc. (Suzhou, China). When the cells reached approximately 50% confluence, they were incubated for 24 hours in a mixture of fresh culture medium, viral solution, and 5 μg/mL of polybrene (C0351; Beyotime Biotechnology). Subsequently, the medium was replaced with fresh medium containing puromycin to select for stably transfected cells.

### Western blot analysis

Western blot analysis was used to determine the levels of proteins associated with mitochondria and mitophagy *in vivo* and *in vitro*. The Bicinchoninic Acid Protein Assay Kit (P0012; Beyotime Biotechnology) was used to quantify proteins. Equal amounts of protein were separated by electrophoresis (150 V, 1.5 hours) using 10% or 12.5% (< 20 kD) sodium dodecyl sulfate gels and electroblotted onto polyvinylidene fluoride membranes (Immobilon-PSQ; EMD Millipore Corporation, Billerica, MA, USA). The membranes were blocked with NcmBlot blocking buffer (P30500; NCM Biotech) for 15 minutes and then probed overnight at 4°C with primary antibodies. The following day, the membranes were incubated for 1 hour at room temperature with the appropriate anti-rabbit or -mouse horseradish peroxidase-conjugated secondary antibodies. After washing three times with Tris-buffered saline/Tween 20, the membranes were incubated with NcmECL Ultra Enhanced Chemiluminescent reagent (P10100; NCM Biotech). Finally, the protein bands were visualized with a chemiluminescence imaging system (4200SF; Tanon Science & Technology Co., Ltd., Shanghai, China) and analyzed using FIJI software (version 2.16.0/1.54p; https://imagej.net/software/fiji/). The catalog numbers and concentrations of the primary and secondary antibodies are shown in **Additional Table 4**.

**Additional Table 4:** Antibodies used in this study

Antibody	CAT# (RRID)	Manufacturer	Dilution	Temperature	Time
anti-P-actin (Mouse)	66009-1-Ig (AB 2687938)	proteintech (Wuhan, China)	1:10000	4°C	Overnight
anti-COX-IV (Rabbit)	AB202554 (AB 2861351)	Abcam (Cambridge, UK)	1:2000	4°C	Overnight
anti-LC3B (Rabbit)	AB192890 (AB 2827794)	Abcam (Cambridge, UK)	1:2000	4°C	Overnight
anti-NeuN (Rabbit)	ab177487 (AB 2532109)	Abcam (Cambridge, UK)	1:250	4°C	Overnight
anti-Parkin (Mouse)	AB77924 (AB 1566559)	Abcam (Cambridge, UK)	1:2000	4°C	Overnight
anti-Rnf144B (Mouse)	MG470696 (AB 3714558)	Abmart (Shanghai, China)	1:2000	4°C	Overnight
anti-SQSTM1/p62 (Rabbit)	AB109012 (AB 2810880)	Abcam (Cambridge, UK)	1:10000	4°C	Overnight
anti-TSPO (Rabbit)	AB109497 (AB 10862345)	Abcam (Cambridge, UK)	1:10000	4°C	Overnight
anti-VDACl (Mouse)	66345-1-Ig (AB 2881725)	proteintech (Wuhan, China)	1:5000	4°C	Overnight
Goat anti-Mouse IgG (H+L) Secondary Antibody, HRP	31430 (AB 228307)	ThermoFisher (MA, USA)	1:10000	Room	1h
Goat anti-Rabbit IgG (H+L) Secondary Antibody, HRP	31460 (AB_228341)	ThermoFisher (MA, USA)	1:10000	Room	1h
Goat Anti-Rabbit IgG H&L (Alexa Fluor® 488)	ab150077 (AB 2630356)	Abcam (Cambridge, UK)	1:100	37°C	1h

COX: Cytochrome c oxidase; HRP: horseradish peroxidase; LC3B: microtubule-associated protein 1 light chain 3 beta; NeuN: neuronal nuclei; Rnf: ring finger protein; TSPO: transport protein; VDAC: voltage dependent anion channel.

After obtaining the grayscale values for the target protein and the corresponding internal reference (COX-IV or β-actin), the ratio of the target blot to the corresponding internal reference was calculated. The data were then imported into Prism 10.4.0 software (GraphPad Software, LLC, San Diego, CA, USA). The unpaired *t*-test was performed for comparisons between the oxyHb and oxyHb + AUTAC4 groups. Comparisons of more than two groups were conducted using one-way analysis of variance, followed by Tukey’s *post-hoc* test.

### Image processing

When analyzing the fluorescence colocalization of different color channels, two images were converted to 8-bit color using FIJI software (version 2.16.0/1.54p; https://imagej.net/software/fiji/). The JACoP plugin was then used to obtain Pearson’s coefficient, M1 and M2, and overlap k1 and k2 data, with Pearson’s coefficient reflecting the colocalization level of different fluorescence channels. FIJI software was also used to analyze the fluorescence intensity of TMRE. The images were converted to 8-bit and inverted. The threshold consistency between groups was controlled before calculating the area, mean gray values, minimum and maximum gray values, integrated density, and threshold limit. Graphic illustrations were created with MedPeer (medpeer.cn) and obtained copyright permission for publication (approval No. 79942thypur9udeqo1744875113 and e9460aqo9rdl1j0cu1752837202, approval date: April 17, 2025 and July 18, 2025).

### Statistical analysis

The normality of continuous variables was tested using the Shapiro–Wilk test before analysis. The unpaired *t*-test was used to compare two groups of normally distributed continuous variables, and one-way analysis of variance, followed by Tukey’s *post hoc* test, was used for multiple comparisons of three or more groups of continuous variables. The data are presented as the mean ± standard deviation. If the data did not follow a normal distribution even after logarithmic transformation, the Kruskal–Wallis *H* test was used, and Tukey’s test was applied for multiple comparisons between groups. The data are expressed as the median (interquartile range). A two-tailed probability (*P*) value of < 0.05 was defined as statistically significant. All statistical analyses were performed using Prism 10.4.0 software (GraphPad Software, LLC, San Diego, CA, USA) or ImageJ 2.16.0 software (https://imagej.net/ij/).

## Results

### Carbonyl cyanide 3-chlorophenylhydrazone induces the collapse of mitochondrial membrane potential in neurons, whereas 10 μM autophagy-targeting chimera 4 reverses the negative effects of carbonyl cyanide 3-chlorophenylhydrazone and activates mitophagy

**Figure 2:**
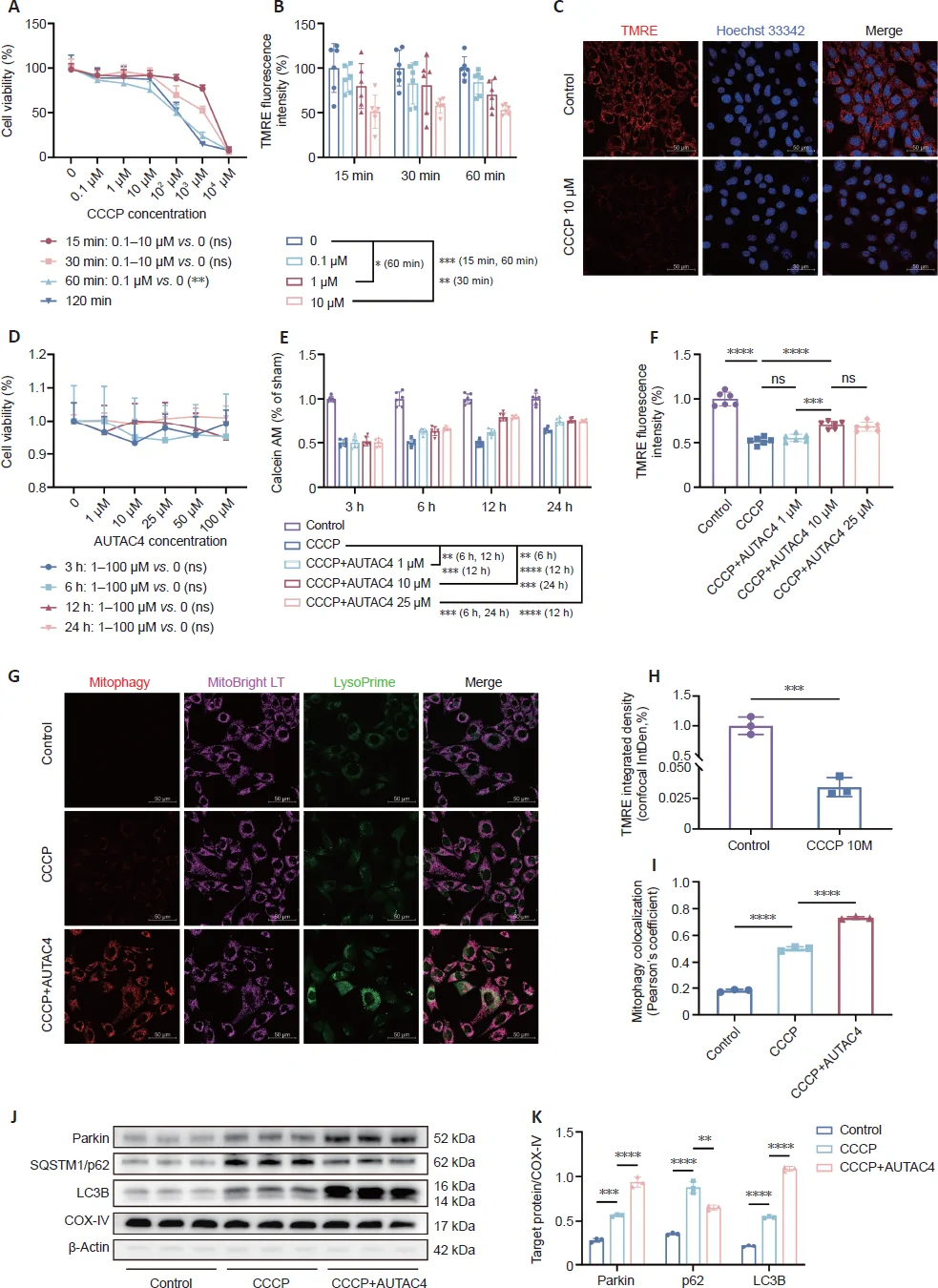
Determination of the dosage and timing of AUTAC4 for the mitochondrial damage model and demonstration of the mitophagic effect. (A) Cell viability at different CCCP concentrations and durations (*n* = 6). (B) The TMRE fluorescence intensity at different CCCP concentrations and durations as detected with a fluorescence microplate reader (*n* = 6). (C) Representative TMRE staining of the control and 10 μM CCCP groups (*n* = 3, scale bars: 50 μm). (D) Cell viability at different AUTAC4 concentrations and durations (*n* = 6). (E) The Calcein AM assay results with different AUTAC4 concentrations and durations (*n* = 6). (F) Under CCCP stimulation, the effects of different concentrations of AUTAC4 on TMRE fluorescence intensity (*n* = 6). (G) Mitophagic staining of the control, CCCP, and CCCP + AUTAC4 groups (*n* = 3, scale bars: 50 μm). (H) Statistical analysis of graph C. (I) Statistical analysis of graph G. (J, K) Representative western blot and quantitative analyses of Parkin, p62, LC3B, and COX-IV (*n* = 3). ns: Not significant; **P < 0.01, ***P < 0.001, ****P < 0.0001 (unpaired *t*-test [H] and one-way analysis of variance followed by Tukey’s *post hoc* test [A, B, D, E, F, I, K]). AUTAC4: Autophagy-targeting chimera 4; CCCP: carbonyl cyanide 3-chlorophenylhydrazone; COX: cytochrome c oxidase; IntDen: integrated density; KD: kilodalton; LC3B: microtubule-associated protein 1 light chain 3 beta; TMRE: tetramethylrhodamine ethyl ester.

Before modeling SAH, we first used CCCP to establish a mitochondrial injury model to determine the dosage and duration of AUTAC4 administration. The CCK8 assay was used to examine the effects of CCCP on the viability of HT22 cells at different concentrations and durations (**Figure 2A**). The results showed that 0.1–10 μM CCCP had no significant effects on cell viability during the stimulation period of 15 to 30 minutes (*P* > 0.05). Fluorescence quantification of TMRE showed that 10 μM CCCP significantly reduced the mitochondrial membrane potential during the stimulation period from 15 to 60 minutes (**Figure 2B**; *P* < 0.001 at 15 and 60 minutes, *P* < 0.01 at 30 minutes). Qualitative results from laser confocal microscopy also confirmed that 10 μM CCCP induced the collapse of the mitochondrial membrane potential in neurons after 30 minutes of stimulation compared with the control group (**Figure 2C** and **H**; *P* < 0.001). Therefore, all subsequent experiments related to CCCP were conducted with 10 μM CCCP stimulation for 30 minutes before detection.

Subsequently, the CCK8 assay demonstrated that 1–100 μM AUTAC4 had no effect on HT22 cell viability within 3 to 24 hours (**Figure 2D**; *P* > 0.05). Furthermore, fluorescence quantification of Calcein AM showed that 1–25 μM AUTAC4 significantly improved the effect of CCCP on cell survival at 6 to 24 hours after administration. The CCCP + AUTAC4 10 μM group exhibited maximum efficacy at 12 hours (*P* < 0.01 at 6 hours, *P* < 0.0001 at 12 hours, *P* < 0.001 at 24 hours), and showed no significant difference compared with the CCCP + AUTAC4 25 μM group (**Figure 2E**; *P* > 0.05 at 3 to 24 hours). This finding was supported by quantitative analysis of TMRE, which showed that the fluorescence intensity in the CCCP + AUTAC4 10 μM group was significantly higher than that in the CCCP group (*P* < 0.0001) and the CCCP + AUTAC4 1 μM group (**Figure 2F**; *P* < 0.001). Therefore, in the subsequent experiments in this section, the concentration of AUTAC4 was set at 10 μM and the duration at 12 hours.

To observe mitophagy, the mitochondria undergoing mitophagy, all mitochondria, and lysosomes were labeled with Mtophagy Dye, MitoBright LT Deep Red, and LysoPrime Green, respectively. Confocal microscopy showed that, compared with the CCCP group, the CCCP + AUTAC4 group had activated mitophagy (**Figure 2G** and **I**; *P* < 0.0001). Additionally, the expression levels of parkin (*P* < 0.001), p62 (*P* < 0.0001), and LC3B (*P* < 0.0001) were significantly increased in the CCCP group compared with the control group, indicating not only an elevated level of mitophagy but also a possible blockage of autophagic flux. Furthermore, the CCCP + AUTAC4 group further increased the expression of parkin (*P* < 0.0001) and LC3B (*P* < 0.0001), and decreased p62 expression (*P* < 0.01) compared with the CCCP group, suggesting that AUTAC4 accelerated the clearance of damaged mitochondria through the selective autophagic pathway (**Figure 2J** and **K**). These results suggest that 10 μM AUTAC4, when administered 12 hours in advance, reversed the negative effects of CCCP on mitochondria and activated mitophagy.

### Activation of mitophagy by autophagy-targeting chimera 4 improved neuronal proliferation and migration after subarachnoid hemorrhage, ultimately reducing neuronal apoptosis

**Figure 3:**
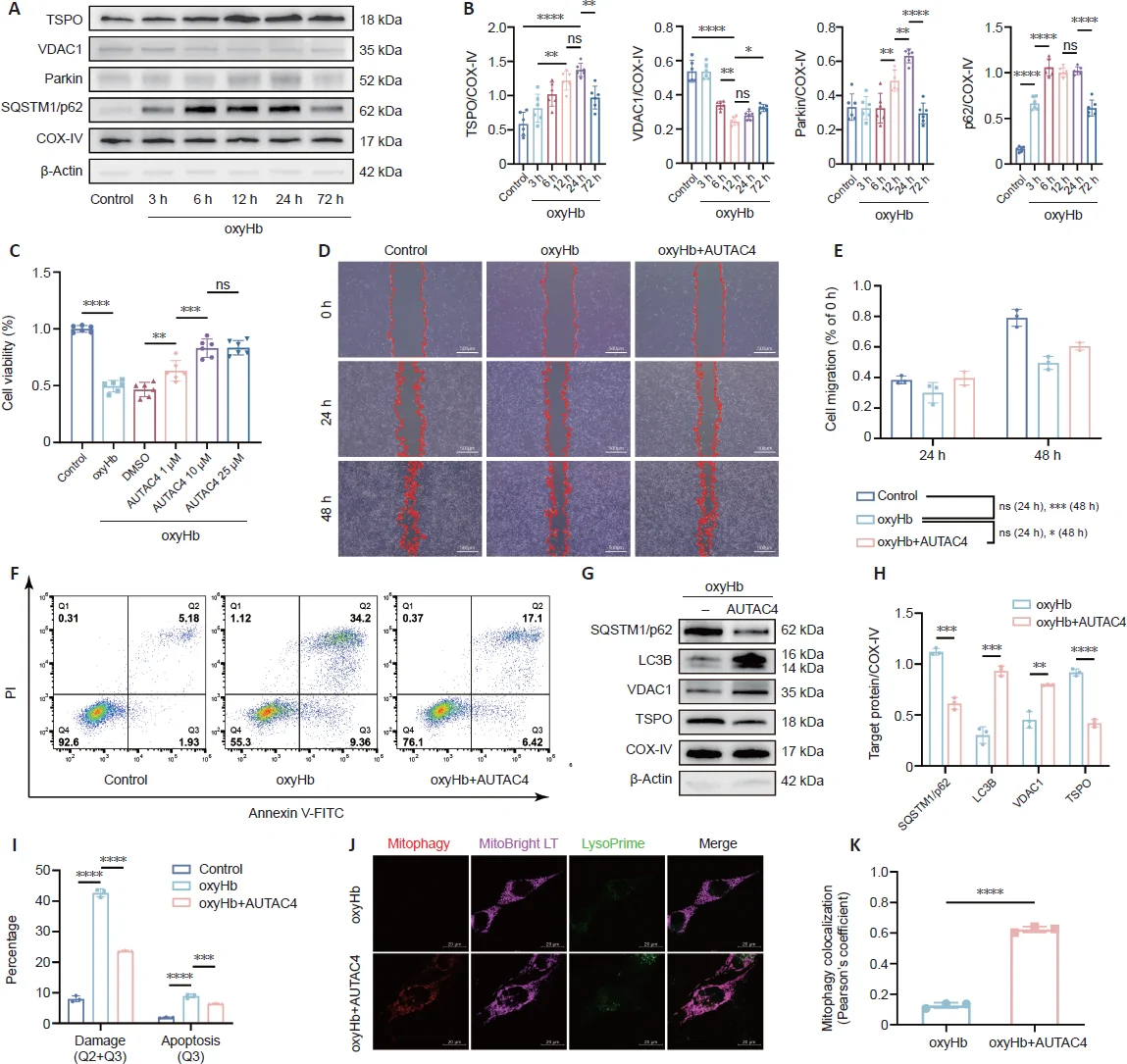
AUTAC4 improves neuronal proliferation and migration after SAH, reducing neuronal apoptosis through mitophagy. (A, B) Representative western blot and quantitative analyses of TSPO, VDAC1, Parkin, p62, COX-IV (*n* = 6). (C) Cell viability at different AUTAC4 concentrations (*n* = 6), (D, E) Cell migration assay of the control, oxyHb, and oxyHb + AUTAC4 groups (*n* = 3, scale bars: 500 μm). (F) The apoptotic rate assessed by flow cytometry (*n* = 3). (G, H) Representative western blot and quantitative analyses of p62, LC3B, VDAC1, TSPO, COX-IV and β-actin of the oxyHb and oxyHb + AUTAC4 group (*n* = 3). (I) Statistical analysis of graph F. (J, K) Representative mitophagic staining and statistical analysis of the oxyHb and oxyHb + AUTAC4 groups (*n* = 3, scale bars: 20 μm). ns: Not significant, *P < 0.05, **P < 0.01, ***P < 0.001, ****P < 0.0001 (unpaired *t*-test [H, K] and one-way analysis of variance followed by Tukey’s *post hoc* test [B, C, E, I]). AUTAC4: Autophagy-targeting chimera 4; COX: cytochrome c oxidase; DMSO: dimethyl sulfoxide; FITC: fluorescein isothiocyanate; KD: kilodalton; LC3B: microtubule-associated protein 1 light chain 3 beta; oxyHb: oxyhemoglobin; PI: propidium iodide; TSPO: transport protein; VDAC: voltage dependent anion channel.

Next, we investigated the protective effects of AUTAC4 in an *in vitro* SAH model, including mitophagy, as well as neuronal proliferation, migration, and apoptosis. First, we confirmed that the oxyHb group (SAH *in vitro*) decreased the mitochondrial membrane potential of neurons (**Additional Figure 1**; *P* < 0.05), as was observed with CCCP. At different time points after SAH, the targets of AUTAC4, TSPO, and voltage dependent anion channel 1 (VDAC1), and the hallmark proteins of mitophagy (parkin and p62) were detected. The expression levels of TSPO (*P* > 0.05 *vs*. oxyHb 12 h, *P* < 0.01 *vs.* oxyHb 72 h), parkin (*P* < 0.01 *vs.* oxyHb 12 h, *P* < 0.0001 *vs*. oxyHb 72 h), and p62 (*P* > 0.05 *vs.* oxyHb 12 h, *P* < 0.0001 *vs.* oxyHb 72 h) all peaked at 24 hours in the oxyHb group, whereas VDAC1 (*P* > 0.05 *vs*. oxyHb 12 h, *P* > 0.05 *vs.* oxyHb 72 h) expression decreased and mitochondrial parkin and VDAC1 expression levels were low (**Figure 3A** and **B**).

Subsequently, the effect of AUTAC4 on neuronal phenotypes after SAH was examined. Compared with the oxyHb group, the oxyHb + AUTAC4 group had increased neuronal proliferation (oxyHb + AUTAC4 10 μM *vs*. oxyHb + AUTAC4 1 μM, *P* < 0.001; oxyHb + AUTAC4 10 μM vs. oxyHb + AUTAC4 25 μM, *P* > 0.05) and migration capabilities (*P* < 0.05 *vs*. oxyHb 48 h) after SAH *in vitro* (**Figure 3C–E**), thereby alleviating neuronal apoptosis (**Figure 3F** and **I**; *P* < 0.0001 at Q2 + Q3, *P* < 0.001 at Q3). Both western blot analysis (p62: *P* < 0.001, LC3B: *P* < 0.001, VDAC1: *P* < 0.01, and TSPO: *P* < 0.0001) and confocal imaging (*P* < 0.0001) indicated that mitophagy was markedly higher in the oxyHb + AUTAC4 group than in the oxyHb group (**Figure 3G**, **H**, **J**, and **K**). Moreover, in both the SAH *in vitro* model and the CCCP model, the application of AUTAC4 significantly inhibited the mitochondrial expression of p62, suggesting that AUTAC4 accelerated the process of mitophagy. These results demonstrated that 10 μM AUTAC4 enhanced neuronal proliferation and migration during the acute phase of SAH by activating mitophagy, ultimately alleviating neuronal apoptosis.

### Autophagy-targeting chimera 4 at 1 mg/kg improved neurological deficits in subarachnoid hemorrhage rats by alleviating neuronal apoptosis

**Figure 4:**
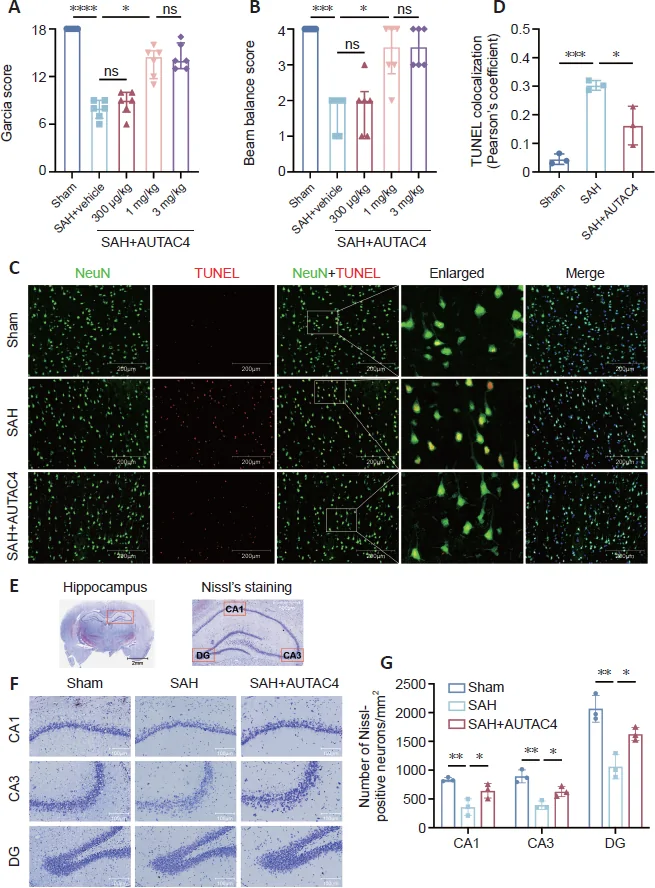
AUTAC4 attenuates neurological deficits after SAH *in vivo* by alleviating neuronal apoptosis. (A, B) Garcia and beam balance scores of each group (*n* = 6). (C, D) Representative TUNEL staining and statistical analysis of each group (*n* = 3, scale bars: 200 μm). (E) Interest areas of the CA1, CA3, and DG regions in the left hippocampus. Black scale bar: 2 mm; white scale bar: 500 μm. (F, G) Representative Nissl staining and statistical analysis of each group (*n* = 3, scale bars: 100 μm). ns: Not significant; *P < 0.05, **P < 0.01, ***P < 0.001, ****P < 0.0001 (Kruskal-Wallis *H* followed by Dunn’s test [A, B] and one-way analysis of variance followed by Tukey’s test [C, G]). AUTAC4: Autophagy-targeting chimera 4; CA: cornu ammonis; DG: dentate gyrus; NeuN: neuronal nuclei; SAH: subarachnoid hemorrhage; TUNEL: terminal deoxynucleotidyl transferase dUTP nick end labeling.

After validating the SAH model *in vitro*, we proceeded to verify the *in vivo* effects of AUTAC4. Rats were intraperitoneally injected with different doses of AUTAC4 (300 μg/kg, 1 mg/kg, and 3 mg/kg) at 12 hours after SAH, and the neurological functions of rats in each group were assessed at 24 hours after SAH. The neurological deficits caused by SAH were significantly improved in the SAH + AUTAC4 1 mg/kg group (*P* < 0.05), and there was no significant difference between the SAH + AUTAC4 1 mg/kg and SAH + AUTAC4 3 mg/kg groups (**Figure 4A** and **B**; *P* > 0.05).

TUNEL staining was used to assess neuronal apoptosis in the sham, SAH, and SAH + AUTAC4 groups at 24 hours after SAH. The results showed that compared with the SAH group, the SAH + AUTAC4 group had decreased neuronal apoptosis (*P* < 0.05), suggesting that AUTAC4 may reduce neurological deficits in SAH rats by alleviating neuronal apoptosis (**Figure 4C** and **D**).

Subsequently, Nissl staining was used to assess long-term neuronal damage after SAH (**Figure 4E**). The results showed significant reductions in Nissl-positive neurons in the CA1, CA3, and dentate gyrus (DG) regions in the SAH group (CA1: 357.7 ± 143.6/mm^2^, CA3: 392.3 ± 73.3/mm^2^, DG: 1060.3 ± 213.1/mm^2^) compared with the sham group (CA1: 835.7 ± 36.5/mm^2^, *P* < 0.01; CA3: 891.3 ± 112.5/mm^2^, *P* < 0.01; DG: 2067.3 ± 233.3/mm^2^, *P* < 0.01; **Figure 4F** and **G**). The SAH + AUTAC4 group showed significantly ameliorated SAH-induced neuronal loss (CA1: 641.3 ± 126.1/mm^2^, *P* < 0.05; CA3: 625.7 ± 86.3/mm^2^, *P* < 0.05; DG: 1625.7 ± 113.1/mm^2^, *P* < 0.05). These results indicated that AUTAC4 not only mitigated short-term neuronal apoptosis and improved neurological deficits in rats, but also reduced long-term neuronal loss.

### Rnf144B was a downstream target of autophagy-targeting chimera 4 in the *in vivo* and *in vitro* subarachnoid hemorrhage models

**Figure 5:**
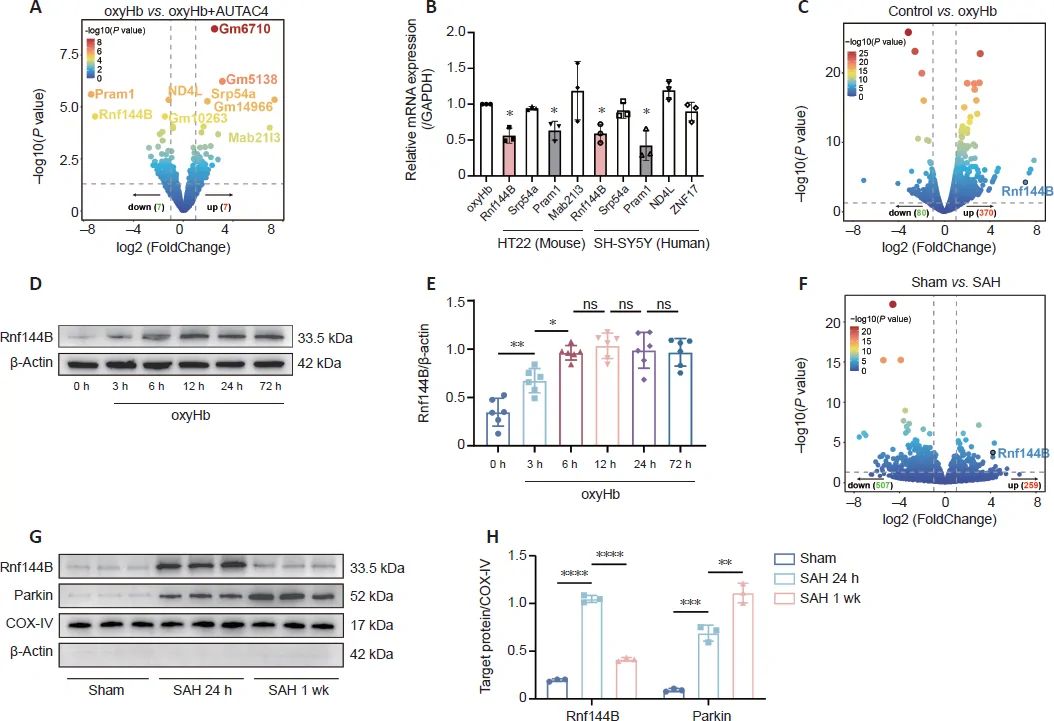
AUTAC4 inhibits the expression of Rnf144B, which is elevated in the *in vivo* and *in vitro* SAH models. (A) RNA-seq between the oxyHb and oxyHb + AUTAC4 groups. (B) qPCR validation of genes screened by RNA-seq (*n* = 3). (C) RNA-seq between the control and oxyHb groups; (D, E) Representative western blot and quantitative analyses of Rnf144B at different time points after SAH *in vitro* (*n* = 6). (F) RNA-seq between the Sham and SAH groups. (G, H) Representative western blot and quantitative analyses of Rnf144B and Parkin at different time points after SAH *in vivo* (*n* = 3). ns: Not significant; *P < 0.05, **P < 0.01, ***P < 0.001, ****P < 0.0001 (unpaired *t*-test [B], one-way analysis of variance followed by Tukey’s test [E, H] and |Log2 fold change| > 1, log10 (*P* value) > 1.3 [A, C, F]). AUTAC4: Autophagy-targeting chimera 4; COX: cytochrome c oxidase; KD: kilodalton; Mab21l3: mab 21 like 3; ND4L: NADH dehydrogenase subunit 4L; oxyHb: oxyhemoglobin; Pram: PML-RARA regulated adaptor molecule; Rnf: ring finger protein; SAH: subarachnoid hemorrhage; Srp: signal recognition particle; ZNF: zinc finger.

To elucidate the mechanism of AUTAC4, we used RNA-seq to screen for differentially expressed genes before and after AUTAC4 administration, followed by validation using qPCR and western blot. The RNA-seq results showed that the oxyHb + AUTAC4 group had seven genes that were upregulated or downregulated compared with the oxyHb group (**Figure 5A**). Subsequently, two neuronal cell lines, HT22 and SH-SY5Y, derived from different species were used for qPCR verification (**Figure 5B**). The qPCR results showed that the expression levels of *Rnf144B* (*P* < 0.05 for HT22 and SH-SY5Y) and *Pram1* (*P* < 0.05 for HT22 and SH-SY5Y) were decreased in the oxyHb + AUTAC4 group, consistent with the RNA-seq results.

The RNA-seq results also showed that the oxyHb group had 370 upregulated and 80 downregulated genes compared with the control group. Notably, *Rnf144B* was significantly upregulated in the oxyHb group (**Figure 5C**). Following SAH, Rnf144B expression was increased after 3 hours of oxyHb stimulation (*P* < 0.01 *vs.* 0 hours), peaked at 6 hours (*P* < 0.05 *vs.* 3 hours), and persisted until 72 hours (*P* > 0.05 *vs*. 24 hours; **Figure 5D** and **E**).

In addition, RNA-seq of rats showed that *Rnf144B* expression was increased in the SAH group compared with the sham group (**Figure 5F**). Subsequently, Rnf144B expression levels at 24 hours and 1 week after SAH were measured. Because parkin and Rnf144B belong to the E3 ubiquitin ligase family, parkin expression was also measured. Rnf144B expression was significantly increased in the SAH 24 h group (*P* < 0.0001 *vs.* sham) and then returned to the pre-modeling level in the SAH 1 wk group (*P* < 0.0001 *vs*. SAH 24 h), whereas parkin expression continued to increase from 24 hours to 1 week after SAH (*P* < 0.001 *vs*. sham; *P* < 0.01 *vs.* SAH 24 h; **Figure 5G** and **H**). These findings indicated that Rnf144B expression significantly increased during the acute phase of SAH and may be associated with poor prognosis.

### Rnf144B is a key node in mitophagy-regulated neuronal apoptosis and interacts with parkin

**Figure 6:**
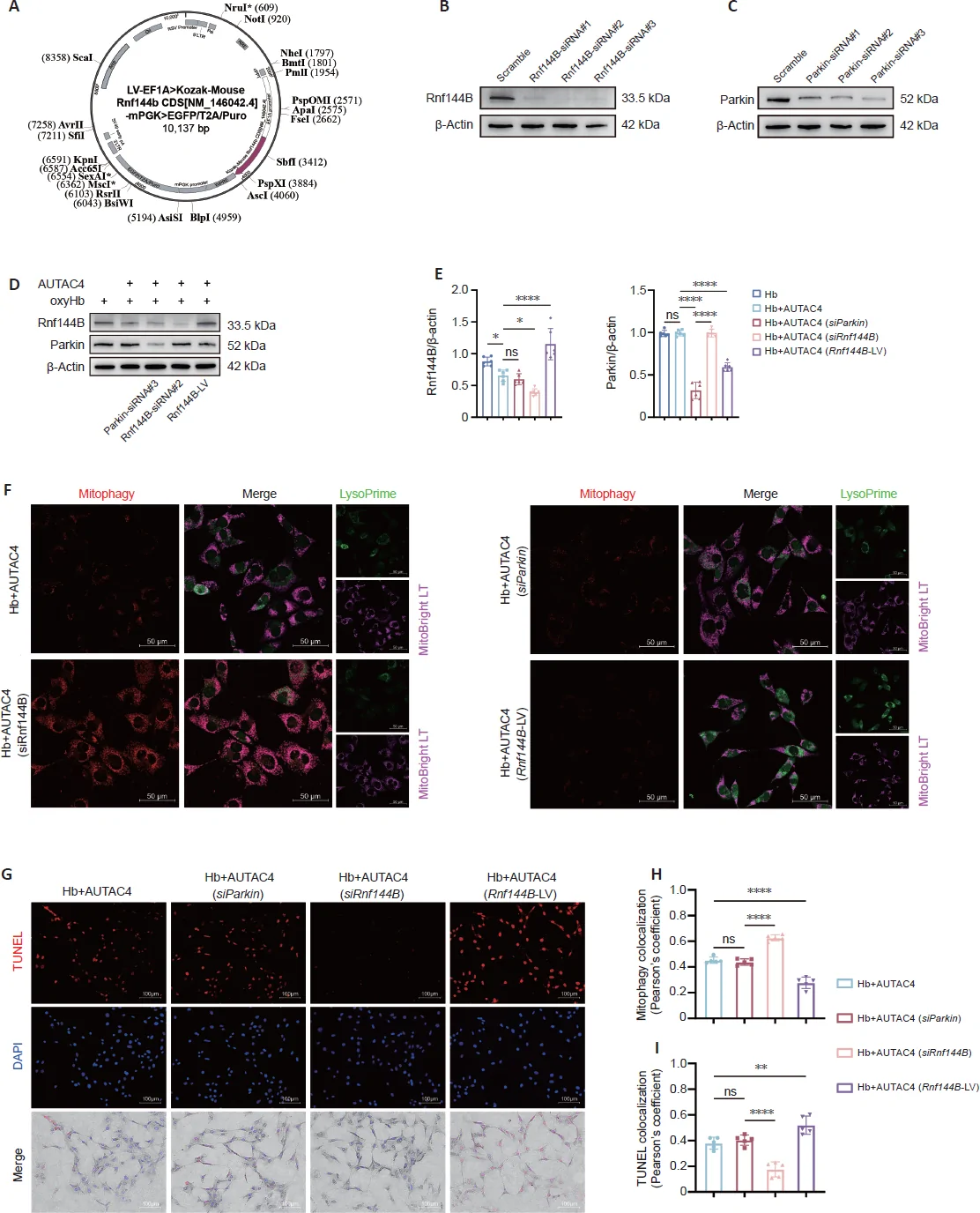
Rnf144B is a key node in AUTAC4-mediated regulation of neuronal apoptosis through mitophagy. (A) Lentiviral vector map for Rnf144B overexpression. (B) Relative levels of Rnf144B in HT22 neuronal cells transfected with Rnf144B-siRNA. (C) Relative levels of Parkin in HT22 neuronal cells transfected with Parkin-siRNA. (D, E) Representative western blot and quantitative analyses of Rnf144B and Parkin in each group (*n* = 6). (F, H) Representative mitophagic staining and statistical analysis of each group (*n* = 5, scale bar: 50 μm). (G, I) Representative TUNEL staining and statistical analysis of each group (*n* = 5, scale bar: 100 μm). ns: Not significant; *P < 0.05, **P < 0.01, ****P < 0.0001 (one-way analysis of variance followed by Tukey’s *post hoc* test). AUTAC4: Autophagy-targeting chimera 4; CDS: coding sequence; DAPI: 4′,6-diamidino-2-phenylindole; EGFP: enhanced green fluorescent protein; KD: kilodalton; LV: lenti-virus; oxyHb: oxyhemoglobin; PGK: phosphoglycerate kinase; Puro: puromycin; RNA: ribonucleic acid; Rnf: ring finger protein; si: siRNA; TUNEL: terminal deoxynucleotidyl transferase dUTP nick end labeling.

To verify that Rnf144B is the key target of AUTAC4-activated mitophagy, lentiviral vectors were used to construct an HT22 cell line with stable overexpression of Rnf144B (**Figure 6A**), and siRNA was used to knockdown *Rnf144B* and *Parkin* (**Figure 6B** and **C**). Then, the modified HT22 cells were continuously stimulated with 10 μM oxyHb for 24 hours, followed by the addition of 10 μM AUTAC4 at 12 hours after oxyHb stimulation, and incubation was continued for 12 hours. Western blotting showed that Rnf144B expression was significantly reduced in the Hb + AUTAC4 group compared with that in the Hb group (*P* < 0.05), and parkin expression was not significantly affected (*P* > 0.05; **Figure 6D** and **E**). Parkin-siRNA#3 significantly inhibited parkin expression (*P* < 0.0001 *vs.* Hb + AUTAC4), and there was no significant change in Rnf144B expression between the Hb + AUTAC4 and Hb + AUTAC4 (*siParkin*) groups (*P* > 0.05; **Figure 6D** and **E**). Similarly, Rnf144B-siRNA#2 knocked down Rnf144B (*P* < 0.05 *vs*. Hb + AUTAC4), and there was no significant difference in parkin expression between the Hb + AUTAC4 and Hb + AUTAC4 (*siRnf144B*) groups (*P* > 0.05). However, the Hb + AUTAC4 (*Rnf144B*-LV) group showed a significant decrease in parkin expression compared with that in the Hb + AUTAC4 group (*P* < 0.0001). Collectively, these results suggested that Rnf144B may interact with parkin at the protein level.

Mitophagic fluorescence staining and TUNEL staining of the four groups of HT22 cells, except for the Hb group (**Figure 6F–I**), showed that compared with the Hb + AUTAC4 group, the Hb + AUTAC4 (*siRnf144B*) group had significantly enhanced mitophagy (*P* < 0.0001) and attenuated neuronal apoptosis (*P* < 0.001) during the short-term period of oxyHb stimulation, whereas the Hb + AUTAC4 (*siParkin*) group did not have significant changes in these measures (*P* > 0.05 *vs*. Hb + AUTAC4). As suspected, the Hb + AUTAC4 (*Rnf144B*-LV) group showed inhibited mitophagy (*P* < 0.0001 *vs.* Hb + AUTAC4) and exacerbated neuronal apoptosis (*P* < 0.01 *vs.* Hb + AUTAC4). These results indicated that during the acute phase of SAH, Rnf144B was a key node regulating mitophagy and apoptosis, and that AUTAC4 suppressed its expression and parkin did not participate in this process.

## Discussion

In this study, we showed that targeting damaged mitochondria for clearance by selective autophagy using AUTAC4 attenuated acute neuronal injury after SAH. Moreover, we identified Rnf144B as a key downstream target of AUTAC4, as its expression rapidly increased during the acute phase following SAH. The increased expression of Rnf144B, both *in vivo* and *in vitro*, was associated with neuronal injury, suggesting that Rnf144B is a critical regulator of mitophagy-mediated neuronal apoptosis in the short term. Additionally, although both parkin and Rnf144B are E3 ubiquitin ligases, AUTAC4 was not affected by parkin knockdown, and overexpression of Rnf144B inhibited parkin expression, demonstrating an interaction between Rnf144B and parkin.

Drugs with mitophagy activity often lack selectivity (Zhang et al., 2019, 2022b; Liu et al., 2023). Recent studies have begun to focus on the development of mitophagy-targeted drugs that minimize off-target effects and to assess the role of mitophagy in mitochondrial quality control (Takahashi et al., 2019; Ding et al., 2020; Mei et al., 2023). As a synthetic chimera, AUTAC4 recognizes the mitochondrial damage marker TSPO at one end and is recognized by the autophagosome LC3 at the other, and directly promotes the clearance of damaged mitochondria via selective autophagic pathways (Takahashi et al., 2019). This recognition mode is more efficient because expression of the E3 ubiquitin ligase Parkin increases slowly after SAH (Cao et al., 2017; Zhang et al., 2019) and may fail to promptly label damaged mitochondria through ubiquitination. This could lead to the accumulation of damaged mitochondria that cannot be cleared via autophagic pathways, thereby exacerbating neuronal damage. TSPO is responsible for the recognition of AUTAC4, and Rnf144B serves as a critical regulator between mitophagy and apoptosis.

Within the E3 ubiquitin ligase family, the most prevalent group is the RING (Really Interesting New Gene) finger (RNF) family, which contains the N-terminal RING domain (Cai et al., 2022). Over 340 RNF members have been identified in humans (Medvar et al., 2016). Studies have shown that the RNF family is involved in various biological processes, and its functional abnormalities can lead to several diseases, including cancer (especially virus-related cancers), immune disorders, and neuropsychiatric diseases (Zhang et al., 2022c; Li et al., 2023a; Yang et al., 2023). Studies have reported that mutations and deletions of RNF family genes, such as the parkin gene and MDM2, result in poor prognosis in gliomas (Veeriah et al., 2010; Sarisozen et al., 2019). A mutation in RNF56 was reported to cause multiple sclerosis (Sanna et al., 2010), and knocking out RNF128 was shown to lead to multiorgan immune dysregulation and significant infiltration of inflammatory cells (Xu et al., 2023; Yu et al., 2024). Various RNF family genes and proteins have also been shown to exhibit abnormal functions in neurodegenerative diseases such as Alzheimer’s disease (characterized by depletion of parkin and overexpression of RNF146 and RNF182) (Liu et al., 2008; Fang et al., 2019; Yi et al., 2024), and Parkinson’s disease (characterized by parkin gene mutations and parkin nitrosylation) (Shimura et al., 2001; Quinn et al., 2020). Ihara et al. (2022) identified RNF213 as the strongest susceptibility gene for moyamoya disease in East Asian populations. RNF213 deficiency activated the Hippo pathway, promoted the overexpression of vascular endothelial growth factor receptor 2, which increased pathological angiogenesis in the cerebral cortex and retina, and ultimately led to moyamoya disease (Ye et al., 2023; Fang et al., 2024). Additionally, in the brain tissue of RNF213 knockout mice, impaired blood–brain barrier function, activated microglia, and elevated levels of proinflammatory cytokines were observed (Li et al., 2023b), underscoring the critical role of the RNF family in the central nervous system.

RNA-seq was used to screen for Rnf144B as a downstream target of AUTAC4, which was further validated by qPCR and western blot analyses. Rnf144B, also known as p53RFP, IBRDC2, or PIR2, belongs to the E3 ubiquitin ligase family, similar to parkin (Li et al., 2024). It is induced by p53 to participate in p53-dependent apoptosis or by p73 to inhibit the antiapoptotic effects of p73 (Sayan et al., 2010; Abad et al., 2024). The role of Rnf144B in disease remains controversial. Some studies have reported that Rnf144B deficiency promotes interferon production, enhancing antiviral immunity in mice (Li et al., 2024), whereas others have shown that Rnf144B deficiency leads to increased production of inflammatory cytokines in response to lipopolysaccharide stimulation, exacerbating cardiac dysfunction and mortality in septic mice (Guo et al., 2023). The results of the present study suggest that Rnf144B causes excessive mitochondrial damage and apoptosis after SAH, and that high expression also inhibits mitophagy. The role of AUTAC4 is to directly initiate mitophagy through TSPO. Subsequently, the clearance of damaged mitochondria reduces the Rnf144B expression, because when cells are stimulated by apoptotic signals, mitochondrial Rnf144B expression increases (Li et al., 2024). As a member of the E3 ubiquitin ligase family (also part of the RNF family), parkin translocates to the mitochondrial outer membrane under phosphatase and tensin homolog-induced putative kinase 1 (PINK1) induction, where it ubiquitinates multiple outer membrane proteins, exerts a positive feedback mechanism, and generates polyubiquitin chains, thereby activating mitophagy (Narendra and Youle, 2024). Because of parkin’s pivotal function in mitophagy, it has been shown to play a protective role in several major diseases, including neurodegenerative disorders, tumor development, and myocardial ischemia–reperfusion injury (Panicker et al., 2022; Sun et al., 2023; Zhao et al., 2024). However, these effects require that parkin has sufficient time to complete the ubiquitination process. The present study showed that in response to SAH *in vitro*, the recruitment of parkin to the mitochondria increased gradually. Also, parkin was consistently increased from 24 hours to 1 week after SAH *in vivo*. These results demonstrated that the classic PINK1/Parkin mitophagy pathway did not provide effective protection during the acute phase after SAH. The distinctly different expression pattern of Rnf144B, another member of the E3 ubiquitin ligase family, suggests a role of Rnf144B in neuronal survival during the acute phase of SAH, as a key regulator of the apoptotic phenotype through mitophagy.

## Limitations

There are some limitations in this study. Although AUTAC4 targets and activates mitophagy, the *in vivo* experiments were insufficient to determine whether AUTAC4 also affects cells other than neurons. Additionally, Rnf144B overexpression inhibited parkin expression, suggesting a possible interaction between these proteins. However, we did not further investigate this potential interaction, and we plan to carry out detailed studies to elucidate the specific mechanisms of this interaction in the future.

## Conclusions

The results of this study suggest that AUTAC4 rapidly activates mitophagy by inhibiting Rnf144B rather than parkin, thereby alleviating acute neuronal injury after SAH. Rnf144B is a key regulator of neuronal apoptosis through mitophagy. Although it belongs to the E3 ubiquitin ligase family like parkin, Rnf144B exhibits a completely different expression pattern after SAH.

## Additional files:

***Additional Figure 1:*** *Mitochondrial membrane potential (MMP) decreased at 24 hours after spontaneous subarachnoid hemorrhage.*

Additional Figure 1 Mitochondrial membrane potential decreased at 24 hours after spontaneous subarachnoid hemorrhage (SAH).
(A) Representative TMRE staining of the control and 10 μM oxyHb groups (*n* = 3; scale bars: 10 μm). (B) Statistical analysis of graph A. *P < 0.05 (unpaired *t*-test). IntDen: Integrated density; oxyHb: oxyhemoglobin; TMRE: tetramethylrhodamine ethyl ester.

***Additional Table 1:*** *Neurological evaluation.*

***Additional Table 2:*** *Primer sequences.*

***Additional Table 3:*** *siRNA sequences.*

***Additional Table 4:*** *Antibodies used in this study.*

## Data Availability

*All relevant data are within the paper and its Additional files.*
